# Multimodal esophageal cancer imaging: establishing data processing techniques and assessing diagnostic sensitivity

**DOI:** 10.1117/1.BIOS.2.2.022704

**Published:** 2025-05-30

**Authors:** Justina Bonaventura, Natzem Lima, Joshua Routh, Aws Alameri, Shivanand Bomman, Bhaskar Banerjee, Hemanth Gavini, Travis Sawyer

**Affiliations:** a University of Arizona, Wyant, College of Optical Sciences, Tucson, Arizona, United States; b Midwestern University, Department of Pathology, Phoenix, Arizona, United States; c University of Arizona, Banner Health, Tucson, Arizona, United States; d University of Arizona, College of Medicine, Tucson, Arizona, United States

**Keywords:** multimodal imaging, autofluorescence, hyperspectral, polarized light, optical coherence tomography

## Abstract

Multimodal optical imaging techniques have generated significant interest for applications such as cancer detection, as combining complementary modalities could broaden the ability to detect early disease changes, as well as address patient-to-patient variability. However, there are challenges in determining how different imaging modalities may or may not complement one another and how best to capitalize on these advantages through computational analysis. We investigate the application of multimodal imaging for the purpose of detecting esophageal cancer, the sixth most deadly cancer in the world. To achieve this, we acquired multimodal optical imaging data—specifically autofluorescence, hyperspectral, polarized light, and optical coherence tomography (OCT)—from fresh human tissue samples obtained during upper endoscopy. Our analysis then addressed three key questions: Which individual modality best differentiates between healthy and cancerous tissues? How can data from these modalities be integrated to maximize discrimination? What computational methods are suitable for analyzing the resulting high-dimensional multimodal datasets? Our findings indicate that polarized light imaging (PLI) exhibits the strongest discriminatory power under these imaging conditions, with potential benefits observed from combining PLI and OCT in a multimodal approach.

Statement of Discovery This work utilizes autofluorescence, hyperspectral, optical coherence tomography, and polarized light imaging systems to study fresh human esophageal samples *ex vivo*, finding various optical features that show significant differences between healthy and cancerous tissue types. Classification algorithms indicate that polarized light imaging shows the most potential for esophageal cancer diagnostics independently and in combination with optical coherence tomography. These results could help guide future endoscopic development for improved cancer screening.

## 1 Introduction

Due to the advances in optical imaging technology, there are now a variety of tools available for biomedical imaging that are sensitive to different biological contrast mechanisms and operate at different spatial scales. Recently, there has been interest in developing so-called multimodal devices, where multiple imaging techniques are combined into a single system. Establishing effective methods for developing multimodal imaging devices is alluring, as the strengths and shortcomings of each technique can be complementary.^1^ For example, working with a system that can image at different scales could be used for locating suspicious regions in a wide field of view followed by higher resolution inspection of those regions.^2^ Likewise, incorporating imaging modes that rely on different contrast mechanisms into a single imaging system could help clinicians get a more complete representation of biological alterations in the tissue. For example, combining modes that rely on the tissue’s structural interactions with light, with those that respond to the changes in the chemical makeup of the tissue, such as increased hemoglobin absorption in areas with high vascularization, could help to characterize what type of structural and/or biochemical changes are being observed.

Endoscopic imaging is considered, as it is often the gold standard for cancer and health screening in the gastrointestinal tract where illumination, available space, and imaging time are limited. Maximizing the amount of relevant diagnostic information gained through a single procedure would be beneficial as endoscopic scans can be expensive and time-consuming. Toward this end, there have been several groups that have incorporated multiple imaging modes into a single endoscopic system.^3^^–^^5^ Kim et al.^3^ combined white light, multispectral, high-frequency ultrasound, and integrated backscattering coefficient imaging into an endoscope; this system was able to identify colorectal cancers at both surface and subsurface levels, using the different imaging modes for different functionalities. Li et al.^4^ combined optical coherence tomography and fluorescence imaging into an endoscope, and with this, they were able to visually differentiate among layers of rectal tissue in rats *in vivo*. Dai et al.^5^ combined photoacoustic imaging, optical coherence tomography, and ultrasound into a single 1-mm-thick endoscope, and with this system, they were able to identify plaques in human arteries *ex vivo*. Due to the spatial limitations of endoscopes, generally, the design and fabrication required to add additional imaging techniques are challenging; beyond this, once the data have been collected, determining how best to handle and visualize multimodal data is also a challenge.

In this paper, we evaluate the potential of four different imaging modes for esophageal cancer detection and assess their potential to be used multimodally in combination. Esophageal cancer is the sixth deadliest worldwide,^6^ with cases on the rise attributed to increased obesity, gastric reflux disease, smoking, and alcohol consumption.^6^^,^^7^ Prognosis worsens when detected late, after the cancer has spread beyond the mucosal and submucosal layers of the esophagus, so early detection is key to improve patient outcomes; however, most patients are asymptomatic, making early detection challenging.^6^^,^^7^ In addition, the miss rate of the screening procedures is much higher for early stages of the cancer, around 34%, compared with later stages, around 4.5%,^8^ motivating the need for better imaging technologies that are sensitive to early disease changes. Due to esophageal cancer’s tendency to metastasize relatively early on, detection of small nodules close to the beginning of disease progression is particularly important.^7^ Treatment of the cancer is typically surgical resection but can be extended to chemotherapy or radiation after the cancer has spread.^7^

Currently, screening is limited to those who are considered to be at higher risk^7^ and is done via upper endoscopy.^7^ The screening standard, white light endoscopy (WLE),^9^ has limited sensitivity especially in the early stages of the cancer.^2^ Early detection is particularly challenging in regions of Barrett’s esophagus, where columnar epithelial tissue starts to grow in place of the typical squamous epithelium.^10^ The sensitivity can be increased through methods including chromoendoscopy which uses dyes that are absorbed into the squamous mucosa to help visualize changes in the functionality of the tissue.^2^^,^^6^ Another technique to increase the sensitivity of WLE is narrow-band imaging (NBI). NBI has been somewhat broadly adapted in clinical practice and employs specific wavelengths in the illumination of the tissue targeting the absorption spectra of hemoglobin to increase the visualization of vascularization in the mucosal surface—this has been found to have a high sensitivity to dysplasia of around 96%.^6^^,^^9^ Most esophageal cancer is not detected until the later stages,^7^ so staging of the cancer is key in determining the course of treatment. Currently, after the initial detection, a positron emission tomography scan can be carried out to stage the cancer.^7^

Beyond this, there have been efforts to investigate alternative imaging modes for detection. Autofluorescence (AF) imaging, which employs filtration of the illumination and detection ends of the endoscope, has been used to detect esophageal cancer in many previous studies,^9^^–^^11^ targeting the loss of collagen in dysplastic tissue growth.^9^ Several studies have found AF is more effective than WLE in identifying tumors in Barrett’s esophagus regions, as well as in identifying esophageal tumors in their early stages.^11^ However, AF can be limited in targeting specific tissue properties and can fall victim to false-positive results.^9^^,^^10^ AF has been considered for use in combination with WLE and NBI as well which was found to decrease the false-positive rate.^9^^,^^10^^,^^12^

Hyperspectral imaging (HSI) has been used in the past to identify gastrointestinal cancers, showing significant differences in the spectra when looking at *in vivo* colorectal tumors in comparison with healthy tissues.^13^ A hyperspectral endoscopy system has been developed for imaging the gastrointestinal tract; this system was used to image human esophageal tissue *ex vivo*, also finding significant differences in the spectra from healthy and cancerous tissues.^14^ Multispectral imaging has also been used to help identify areas of high-grade dysplasia among Barrett’s esophagus regions with 90% accuracy.^15^ A spectral endoscopy system developed to image esophageal tissue *in vivo* has been able to differentiate between Barrett’s esophagus regions and neoplasia with 84% accuracy.^16^ When incorporated into a multimodal endoscope, multispectral imaging showed several wavelength bands to have statistical significance in comparing healthy and cancerous colorectal cancer samples.^3^ Another work has investigated hyperspectral endoscope systems for imaging colorectal tissue *in vivo* and found significant differences in the spectra in similar wavelength ranges^13^ and with the aid of machine learning algorithms.^17^

Optical coherence tomography (OCT) uses interferometric techniques to provide depth-resolved images of samples up to a few millimeters deep. OCT systems typically have relatively high resolution and are used to probe structural differences in the tissue that occur with tumor growth.^9^ The ability to get tomography-resolved scans of the tissue is promising, as knowledge of how deep the cancer has proliferated is key to guiding treatment.^3^ OCT has been used in esophageal cancer detection studies in the past and was found to have a sensitivity of 83% and a specificity of 75%.^9^ Systems have been brought to market for the purposes of esophageal cancer detection previously by NinePoint Medical,^2^^,^^15^ but determining specific diagnosis criteria from image data remains an active area of research.^2^ The systems brought to market have yet to be broadly adopted.

The potential for polarized light imaging (PLI) to be used for cancer detection has been investigated in a variety of other studies, in several cases for cervical cancer^18^^–^^20^ as well as oral pre-cancer.^21^ PLI has been found to be sensitive to changes in the morphology of microstructural changes,^22^^,^^23^ which shows promise for detecting the chaotic growth tissue undergoes with tumor development. Retardance and depolarization are typically the polarization properties found to show sensitivity to these microstructural changes.^21^^,^^23^ In addition, polarization sensitivity has been combined with other imaging modes previously such as polarization-sensitive OCT and polarization-sensitive spectroscopy^1^^,^^24^ through the use of polarization filtration and sequential imaging techniques.

In this study, the merits of these four imaging techniques for identifying esophageal cancer are assessed individually and then in combination. The overall aim of this work is to explore the potential and feasibility of combining these different imaging types for use in endoscopic esophageal cancer screening. In this case, AF, HSI, OCT, and PLI are investigated independently as well as their potential to be used together in a multimodal system. To do so, we collect imaging data using benchtop systems for fresh healthy, metaplastic, and cancerous tissue samples collected from patients undergoing upper endoscopy procedures. With this dataset, image features are extracted from each modality, and the features with the highest discriminatory ability between healthy and cancerous tissues for each imaging mode are identified. The data are then fed into a classification pipeline to determine how well different feature sets differentiate among the tissue classes, both individually and in combination. Through this, we aim to establish methods for analyzing high-dimensionality datasets associated with multimodal approaches.

The selection of imaging modalities for this study was driven by the complementary contrast mechanisms that each provides and also the compatibility with wide-field endoscopic architectures. OCT produces depth-resolved microstructural information through backscattering,^25^ and PLI complements this with microstructural orientation feature sensitivity.^26^ AF contrast is produced by the abundance of endogenous fluorophores,^27^ and our implementation of HSI measures wavelength-resolved tissue scattering and absorption.^28^ Although the contrast sources for each modality may have some overlap with one another, each system primarily isolates a different mechanism. It should be acknowledged that *ex vivo* tissues may not fully recapitulate *in vivo* characteristics, particularly for HSI and AF, which are often strongly influenced by blood. However, many sources of AF and HSI contrast will still be present in *ex vivo* tissues, for example, collagen or immune cell fluorescence and absorption by lipids or melanin, and this dataset provides an excellent foundation to establish our data analysis and classification pipelines.

## 2 Methods

### 2.1 Sample Collection

**Fig. 1:**
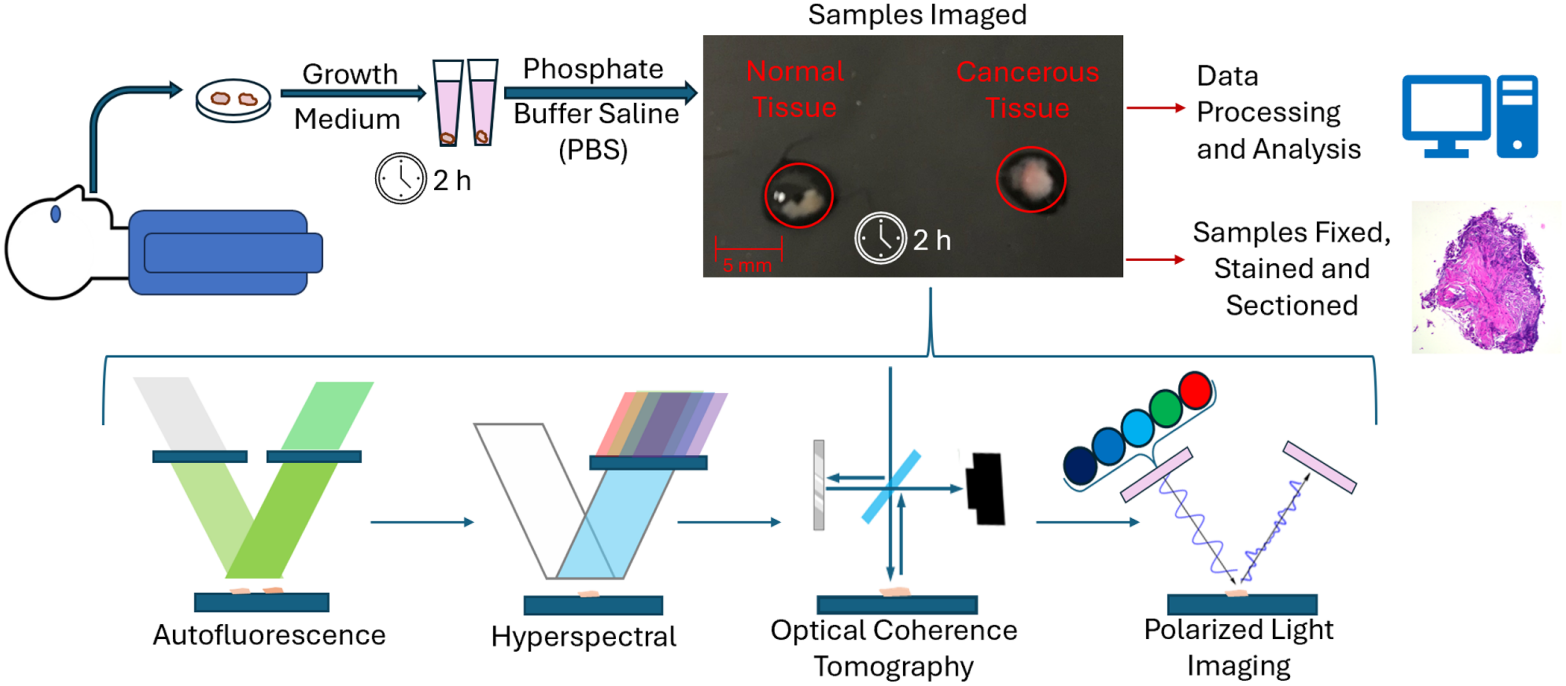
Overview of the sample extraction and imaging process.

As shown in Fig. 1, samples are collected from patients undergoing upper endoscopy procedures for suspected cancer at the Banner-University Medical Center. All but one of the samples were collected as biopsies during the endoscopy using jumbo forceps; a single sample was collected from an esophageal resection. No significant difference in imaging features was observed comparing the resection to the biopsies, so all specimens were included in the dataset for subsequent analysis and classification. The criterion for inclusion was any cancer of the esophagus or esophageal–gastric junction. All human samples were obtained with informed patient consent under IRB Approval No. 1909985869A007. When possible, specimens of healthy, suspected cancerous and Barrett’s esophagus tissue (metaplasia) were collected from each patient to enable direct comparisons, whereas there were some slight variations in the size of the samples, and they were typically 3 to 4 mm in diameter. The single specimen collected from endoscopic resection was ∼10  mm in diameter.

The excised samples were then submerged in Dulbecco’s modified Eagle medium solution and placed on ice to be transported from the clinic to the imaging location to maintain the integrity of cellular biomarkers. For imaging, the samples are transferred to a petri dish without a coverslip and kept moist with a thin layer of a phosphate buffer solution. Specimens were positioned to the best of our abilities to ensure the epithelial side was oriented toward the imaging systems. After imaging, specimens are fixed in paraformaldehyde for ∼24  h, before dehydration in ethyl alcohol solution. Specimens were then embedded in paraffin, sectioned, and stained with hematoxylin and eosin (H&E). These were subsequently assessed by a pathologist for ground truth disease state. A total of 45 samples were collected from 26 patients, 20 of which were normal tissue, 14 were cancerous, and 11 were metaplastic; among those, six were Barrett’s esophagus tissue. Seven of the samples could not be identified by the pathologist, for those we deferred to the clinicians’ initial classification. The full breakdown of tissue types can be reviewed in Table 2 in the Appendix.

### 2.2 Imaging

**Fig. 2:**
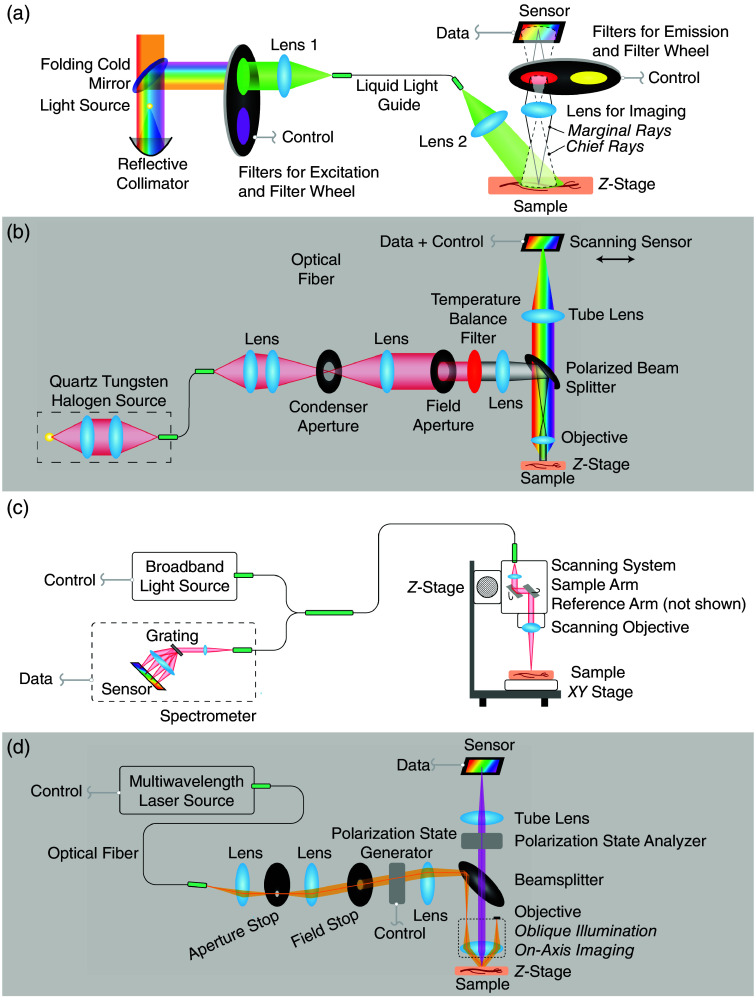
Imaging systems and design layout for (a) autofluorescence imaging system, (b) hyperspectral imaging system, (c) optical coherence tomography system, and (d) polarized light imaging system (Mueller matrix polarimeter system).

The samples are then imaged with the AF, HSI, OCT, and PLI systems. Example images can be seen from each in Fig. 11(a) for healthy and Fig. 11(b) for cancerous, esophageal tissue samples from the same patient. The specifications of the four imaging systems used are summarized in Table 1. For each system and each specimen, the focus depth was manually adjusted until the image was sharply in focus prior to the collection of any data. The samples were first imaged with the AF system, which has been previously described in detail.^29^ As laid out in Fig. 2(a), the AF system uses a broadband Lambda LS Xenon Arc Lamp light source filtered through narrowband filters to image with 340-, 400-, 460-, 490-, and 647-nm illumination in sequence. The samples are imaged in a backscattering configuration, and the system employs filtration on the detection end to isolate specific autofluorescent bands. The system collects images at each illumination wavelength with long-pass detection filters featuring cut-on wavelengths of 380, 430, 500, 532, 561, 594, and 647 nm. The excitation and emission filter wavelengths were selected to target common tissue biomarkers such as nicotinamide-adenine dinucleotide, porphyrin, lipofuscin, and flavin-adenine dinucleotide. The AF system uses a large field of view of 24×36  mm so that the samples can be imaged simultaneously to limit the effects of tissue degradation on the measured autofluorescence. For calibration purposes, dark and flat field images are collected at the start of each imaging session and used to pre-process the data.

**Table 1:** Imaging mode specifications.

Imaging system	Field of view (mm)	Resolution (μm)	Imaging time
Autofluorescence	24 × 36	12.5	10 min
Hyperspectral	2.2 × 2.6	4	2 min
Optical coherence tomography	9 × 9 × 2.6	12 × 3.5	10 s
Polarized light imaging	3 × 3	10	10 min

The samples are next imaged with a hyperspectral scanning sensor camera (Imec, VNIR, Leuven, Belgium), shown in Fig. 2(b), which collects full images across 150 spectral bands ranging from 470 to 900 nm. The system uses a quartz tungsten halogen light source (Thorlabs, OSL2, Newton, New Jersey, United States) with a Kohler illumination configuration for uniform illumination at the sample plane. A wire grid polarizer is used as the polarized beam splitter to reduce specular reflections from the sample. After the light returns from the sample, only light that has interacted with the sample enough to have its polarization altered will be transmitted through the beam splitter, and these techniques are employed to help limit the contributions of specular reflections on the measured spectra. The light from the sample goes through a 40-mm focal length apochromatic objective lens (Thorlabs MY5X-822) and a 200-mm focal length achromatic tube lens (Thorlabs AC254-200-AB-ML) arranged in a 5× magnification configuration. Prior to imaging, a white calibration target is imaged so that the spectral dependence of the illumination system can be removed from the sample spectra. The samples are imaged sequentially, first the healthy and then the cancerous tissue.

Following this, a Thorlabs Telesto OCT system (Thorlabs TEL221C1) [Fig. 2(c)] was used to take depth-resolved images of the samples. This system functions as an interferometer at each point in the horizontal plane of the sample by reflecting infrared light with a central wavelength of 1300 and a 170-nm bandwidth into the sample. The variations in wavelength across the reflected light are used to reconstruct the profile of the sample in the vertical direction with a depth of up to 2.6 mm. The samples are once again imaged sequentially with the healthy sample first.

Finally, the samples are imaged with a Nikon full Mueller backscattering polarimeter at 405, 442, 473, 543, and 632 nm [Fig. 2(d)]. This system is described in detail in previous publications^30^^,^^31^ and uses a linear polarizer and quarter-wave plate to sequentially generate desired polarization states for illumination. After the light is backscattered from the sample, the polarization state analyzer employs a pair of Savart plates to encode the intensities of the measured polarized light into spatial frequencies, which can then be separated through Fourier frequency filtering to measure all four Stokes parameters in a single image, as described by Oka and Saito^31^ For each wavelength, the sample is imaged using six different illumination polarization states to calculate the full Mueller matrix via a data reduction algorithm.^32^ After taking a series of calibration measurements using a diffuse reflection target and a linear polarizer to separate the polarization dependencies of the system from the tissue, the samples are imaged sequentially.

### 2.3 Data Processing

#### 2.3.1 Feature extraction

**Fig. 3:**
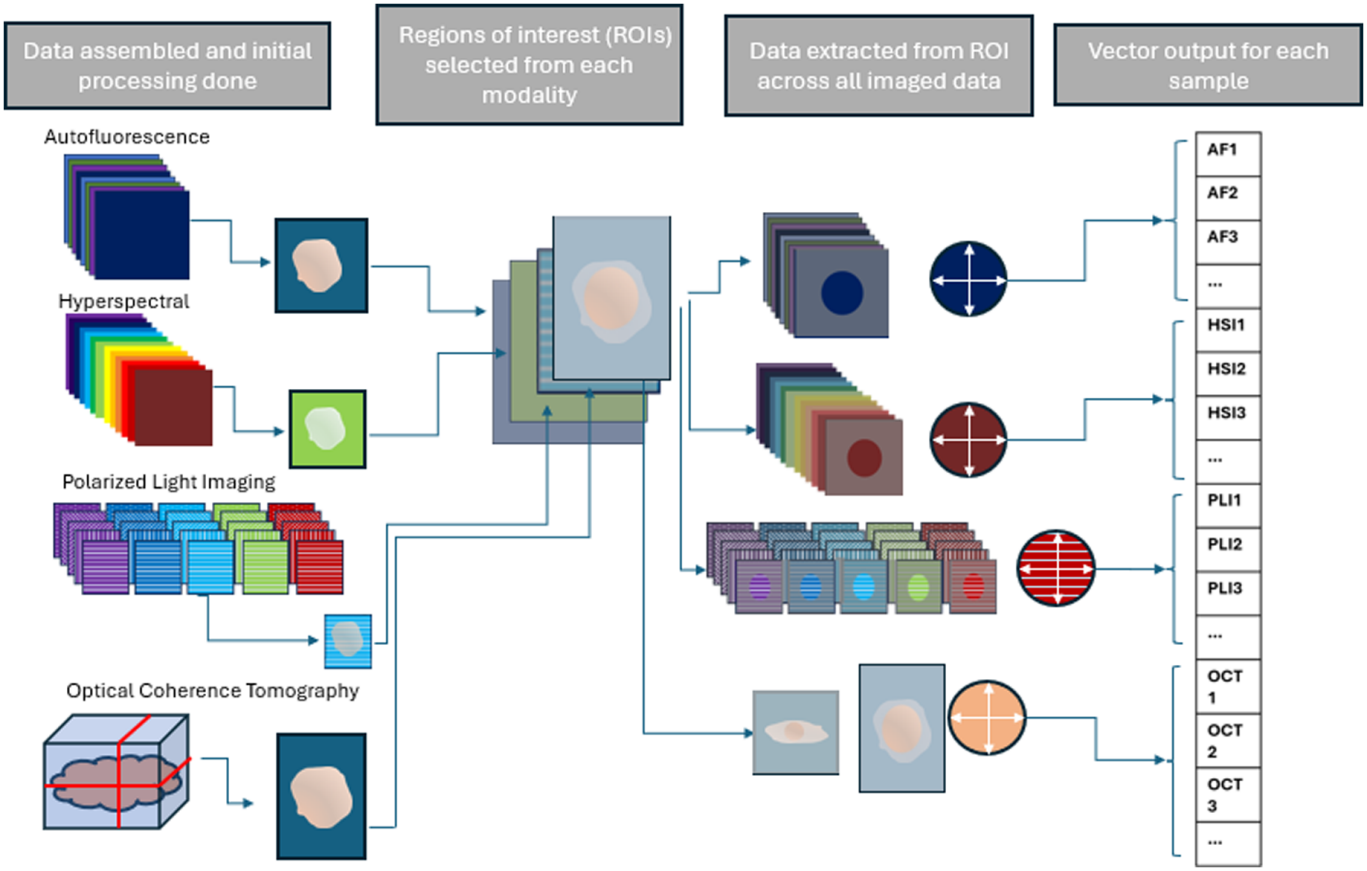
Data processing overview for each sample in which data collected from four imaging modes is assembled, ROIs are selected for each imaging type individually, features are extracted across ROI, and feature output is organized into a single vector for each sample.

An overview of the image feature extraction process is laid out in Fig. 3. First, any potential over-saturation in the collected images was eliminated by removing any pixels with a brightness within 5% of the detector capacity threshold for each system. Then, for each imaging type, circular regions of interest (ROIs) were manually selected with a radius chosen to maximize the image region entirely comprised of tissue and free of background or artifacts. The ROIs were selected for each sample and imaging type independently to allow for analysis across consistent regions of the samples. From here, image features were extracted and averaged over the whole ROI. Spatially averaging features across ROIs negates the necessity of co-registration across the imaging modalities, which allows for each modality to be compared with ease despite having different data structures.

For the autofluorescence images, a flat field correction was first applied. As shown in Fig. 12, the features are then extracted over the ROI: the mean and standard deviation were taken for each excitation and emission filter image [Fig. 12(a)]. For each illumination wavelength, the mean of the images with higher bandpass emission filters is subtracted from those with lower bandpass emission filters to simulate narrowband imaging [Fig. 12(b)]. These narrowband values for each illumination wavelength are then assembled into a pseudo-spectrum by interpolating them in spectral order over the wavelength range they occupy. The phasor approach to spectral analysis,^33^ a fast Fourier transform (FFT) method, is applied to these pseudo-spectra, and the real and imaginary part of the first frequency term are taken as features, as well as the real component of the zeroth order intensity term, referred to as G, S, and I [Fig. 12(c)].

An illumination spectral correction was applied to the HSI data within the Imec software before further processing. From here, the features are extracted; first, the mean and standard deviation were taken across the ROI. The means of each sample’s spectra were then normalized to fit between 0 and 1 due to large variations in spectral range in different imaging sessions [Fig. 13(a)]. Prior to normalization, the ROI means were averaged over 10-nm wavelength ranges to preserve some indication of the original value range of the spectra [Fig. 13(b)]. Similar to the autofluorescence data, the phasor approach is adapted by taking the real and imaginary components of the first- and second-order frequency terms and the real component of the 0^th^-order term, referred to as G1, S1, G2, S2, and I in Sec. 3 and figures [Fig. 13(c)]. To capture the differences in the overall spectral shape among the tissue types, a fourth-order polynomial fit was applied to the normalized spectra taken for each sample—the fit coefficients were taken as features [Fig. 13(d)]. In addition, a Fourier fit was applied to the spectrum as well, and the modulus of the first 10 frequency terms from the FFT was taken as a feature [Fig. 13(e)].

For the OCT datasets, as shown in Fig. 14(a), horizontal and vertical slices were manually selected to maximize the area of high-quality image data, and an ROI was selected within those slices. Haralick texture features^34^ were calculated in the ROIs for both horizontal and vertical slices; the average value over the four directions was computed for each Haralick feature. This was implemented using the Mahotas Python library.^35^ To detect image differences on a broader scale, the FFT was performed on each sample; for this analysis, the ROI was constrained to be the same size among all samples to ensure a constant field of view and pixel spacing during analysis [Fig. 14(b)]. Frequency features were calculated by taking the mean over annular regions within the FFT radiating out from the center. Finally, to consider the depth resolution of the tomography dataset, the attenuation was calculated over the image block to assess the density of the samples. This was done by assembling vectors from the means of each horizontal 10×10  pixel square through the depth profile of the sample and applying thresholding to isolate the measured signal from the tissue. Each profile was fit to an exponential decay curve to calculate the average attenuation coefficient of the whole sample according to Beer’s law [Fig. 14(c)].

For PLI data, the Mueller matrix is first calculated in custom software developed for the microscope by Nikon. This software has built-in sensitivity to numerical instabilities and imaging artifacts which will on occasion result in an inability to calculate the Mueller matrix for datasets from certain wavelengths. Once the Mueller matrix is obtained [Fig. 15(a)], the Lu Chipman decomposition is applied,^36^ which deconstructs the Mueller matrix into individual matrices for retardance, depolarization, and diattenuation, then further into polarization properties such as linear retardance and polarizance. The mean and standard deviation were calculated for all polarization properties across ROIs [Fig. 15(b)], as well as the means of the individual Mueller matrix components for each wavelength. For this dataset, out of the 45 samples, there were six for 632 nm, zero for 543 nm, one for 473 nm, zero for 442 nm, and eight for 405 nm where the Mueller matrix could not be generated; in addition, there were two samples for which the PLI system was not available for imaging due to calibration errors, one Barrett’s, and one cancer sample.

The combined feature outputs for all four imaging modes are organized into a vector of 674 features for each sample, which can then be further analyzed and classified. For some specimens, the feature vectors were incomplete—for example, when the Mueller matrix was not able to be generated. In these circumstances, the specimens are omitted from statistical testing. For classification tasks and feature ranking procedures, the missing data points are interpolated with the means of the other values of that feature from that class. Both the statistical testing and classification procedures are discussed below.

#### 2.3.2 Correlation reduction

**Fig. 4:**
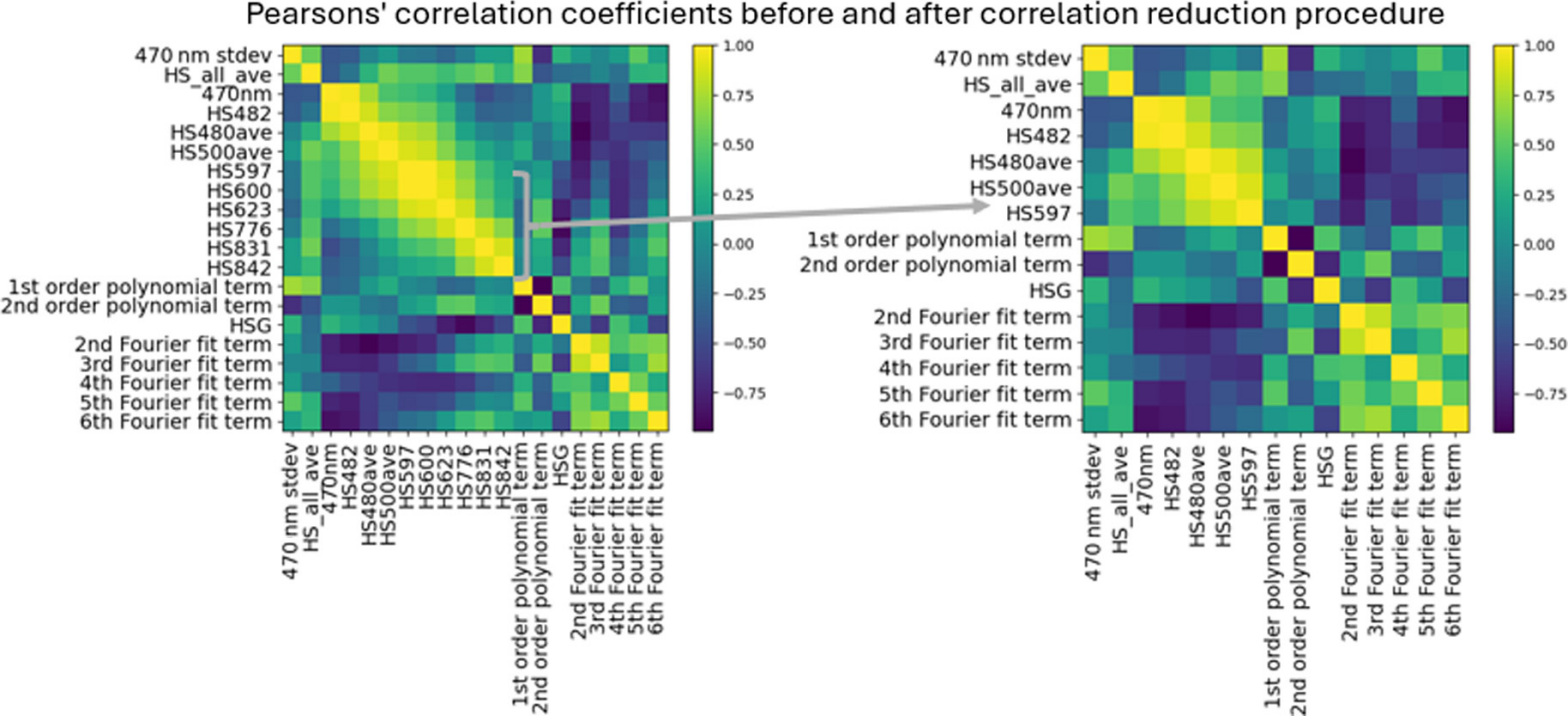
Example of reduction of feature number for highly correlated features in hyperspectral data.

Several considerations need to be made when working with such high-dimensionality data. First, a large number of the features were highly correlated with one another, implying that they measure similar characteristics of the tissue. To reduce redundancy, a correlation reduction procedure was applied as described previously by Sawyer et al.^25^ An example of this is shown in Fig. 4. Prior to doing so, a preliminary Wilcoxon-paired statistical test was carried out to provide p-values that act as a ranking criteria for determining which of the features are best at differentiating among the tissue types. The correlation reduction is an iterative procedure that then takes the Pearson correlation coefficients across all the features. For highly correlated (>0.85) features, the p-values of the two features are compared, and the one with the higher p-value is removed. This process is repeated until the only remaining features possess correlation coefficients below the threshold of 0.85 relative to one another. This procedure was carried out for each imaging type individually and reduces the number of features from 674 to 145 total: from 90 to 18 for AF, 365 to 12 for HSI, 49 to 18 for OCT, and 170 to 95 for PLI.

#### 2.3.3 Statistical analysis

To assess which features have significant differences between the healthy and cancerous specimens, there are a variety of tests that can be applied. First, to determine which approach is most appropriate, a Shapiro–Wilk test was done to assess the normality of the distribution for each feature. To do so, the test was applied to the data separated by feature and class. Through this, it was determined only ∼20% of the features were normally distributed for both healthy and cancerous datasets, so traditional parametric statistical methods are likely not an appropriate approach, and we proceeded with nonparametric methods.

Another factor of note is that for some patients, all tissue types were able to be collected; however, in other cases, only one tissue type was available, producing a dataset with mixed paired and unpaired specimens. There are relevant questions in distinguishing among the tissue types both in the context of within a single patient and across the tissue characteristics more broadly. To probe both these questions, the data were organized into a paired set composed of only the data from patients where there are samples from both healthy and cancerous tissue types and an unpaired set where all the data were pooled together and separated only by class. A Wilcoxon test was done to compare the paired set, and a Mann–Whitney U-test was carried out to compare the unpaired set. These were selected as nonparametric analogs for the traditional T-test. Both of these tests have meaningful implications as the unpaired test is more directly applicable to how the classifiers described below operate, and the paired test allows for comparisons within patient datasets directly which may be more similar to how a clinician would use a tool such as this, i.e., comparing suspicious areas to normal neighboring tissue; however, it is of note that there were a smaller number of paired samples with both cancerous and normal tissue only being collected from eight patients.

The large number of features also requires us to consider how to adjust our p-values from these statistical tests for multiple comparisons as the probability of false positives gets higher as more tests are performed. After the correlation reduction process, the p-values of the remaining features are adjusted using the false discovery rate method with a Benjamini–Hochberg correction. This was done separately for each imaging type with a family-wise error rate (FWER) set to 0.1 in this case. Benjamani–Hoochberg was chosen in this case as it is a fairly standard correction method that is frequently used in exploratory studies such as in genomics. The FWER can be adjusted to be more stringent in terms of rejecting false positives by adjusting the FWER to be lower; however, for this study, our primary goal is the identification of potentially valuable image features, and therefore, the reduction of false negatives was balanced with false positives. However, these limitations should be kept in mind in the interpretation of our results, suggesting that further validation studies should be performed on features that show high statistical significance.

#### 2.3.4 Classification and feature ranking

A leave-one-out method was used to separate the data into test and training sets iteratively. A random forest (RF) classifier was used because this model does not make assumptions about the distribution of the data and random forest classifiers work well with high-dimensional data.^37^ In addition, linear discriminant analysis (LDA) was performed using the leave-one-out approach, as this is also a standard classification approach with high-dimensionality data and limited sample sizes. Receiver operating characteristic (ROC) curves were generated to assess the performance of the two classifiers, with the area under the curve (AUC) acting as the metric for how well the classifiers perform. With the leave-one-out method, probability estimates are generated for how likely the classifier is to correctly identify the test sample set aside for each iteration, and the ROC and AUC calculations are then made from this set of accumulated probabilities across all test samples.

As the number of features used with these classifier models has been shown to have a large impact on their performance;^25^ these classifiers were run using the 10 best features and the 3 best features as determined by the Gini importance method for the RF classifier and the scatter coefficient ratio method for LDA, both described below. To compare how each imaging type performs individually, this procedure was carried out using the top features from each of the four imaging modalities first and then by pooling them together to find the top features with the multimodal dataset. The majority of the analysis in this report focuses on comparing healthy and cancerous samples; however, this classification procedure was also run comparing other tissue types. The random forest classifier using 10 features performed the best in the majority of cases, so the results shown were calculated accordingly. In this way, we compare healthy and abnormal tissues, i.e., cancerous and metaplastic tissue types pooled together shown in S6a. Classification procedures were also run for healthy and metaplastic tissue alone (S6b) and metaplastic and cancerous tissue (S6c).

In determining which features were most relevant to separating between the healthy and cancerous tissue types, two techniques were employed. First, as much of the data was not normally distributed, Gini importance (GI) calculations were performed. GI calculations measure how much each feature will set apart the two tissue types for randomized subsets of the data. This was calculated using the feature importance property of the scikit learn random forest classifier. GI is a commonly used criterion for random forest generation as suggested by Breiman et al.^38^ and is typically defined for each feature as

$${\mathrm{GI}}_{F}=\overset{D}{\underset{k}{\sum }}\frac{n_{k}}{n}\overset{C}{\underset{i}{\sum }}p_{i}^{2}.$$ (1)

Here, C is the total number of classes, D is the number of branches the feature is divided up into, $n_{k}$ is the number of samples in that subdivision, n is the total number of samples, and $p_{i}$ is the probability of samples in that subdivision belonging to the class i.

Second, scatter coefficients, scatter within $S_{w}$ and scatter between $S_{B}$, were calculated for each feature to assess the variance in the datasets between and within the two tissue types, adapting the methods described in Ref. 25. $S_{w}$ and $S_{B}$ are defined accordingly,

$$S_{w}=\sum_{i}\sum_{j}(x_{j}-m_{i})(x_{j}-m_{i}{)}^{T}.$$ (2)

Here, i is the class, j is the patient dataset, $m_{i}$ is a vector made up of the means of all patient data for each feature, and $x_{j}$ is a vector of all features for each patient dataset,

$$S_{B}=\sum_{i}n_{i}(m_{i}-m)(m_{i}-m{)}^{T}.$$ (3)

In $S_{B}$, $n_{i}$ is the number of features in each class, $m_{i}$ is again a vector of means for each class, and m is a vector of means taken for all patient data for each feature. The ability for each feature to differentiate between the two classes will be highest when $S_{w}$ is at a minimum and $S_{b}$ is at a maximum, so the ratio of $S_{b}$ and $S_{w}$ will act as a metric for feature relevance. In this case, the three features with the highest ratio values are taken to be the top features by this method.

In the feature of interest plots that follow in the Sec. 3, features that suggest statistical significance with paired significance are denoted by stars and unpaired significance denoted by triangles, two of each symbol are indicative of an adjusted p-value below 0.05 and one of each symbol of an adjusted p-value below 0.1, the colored bars on the sides of the plots represent top features as determined by the Gini importance in green and the scattering coefficients in purple, with darkness of hue indicating higher ranking.

## 3 Results and Discussion

### 3.1 Autofluorescence

**Fig. 5:**
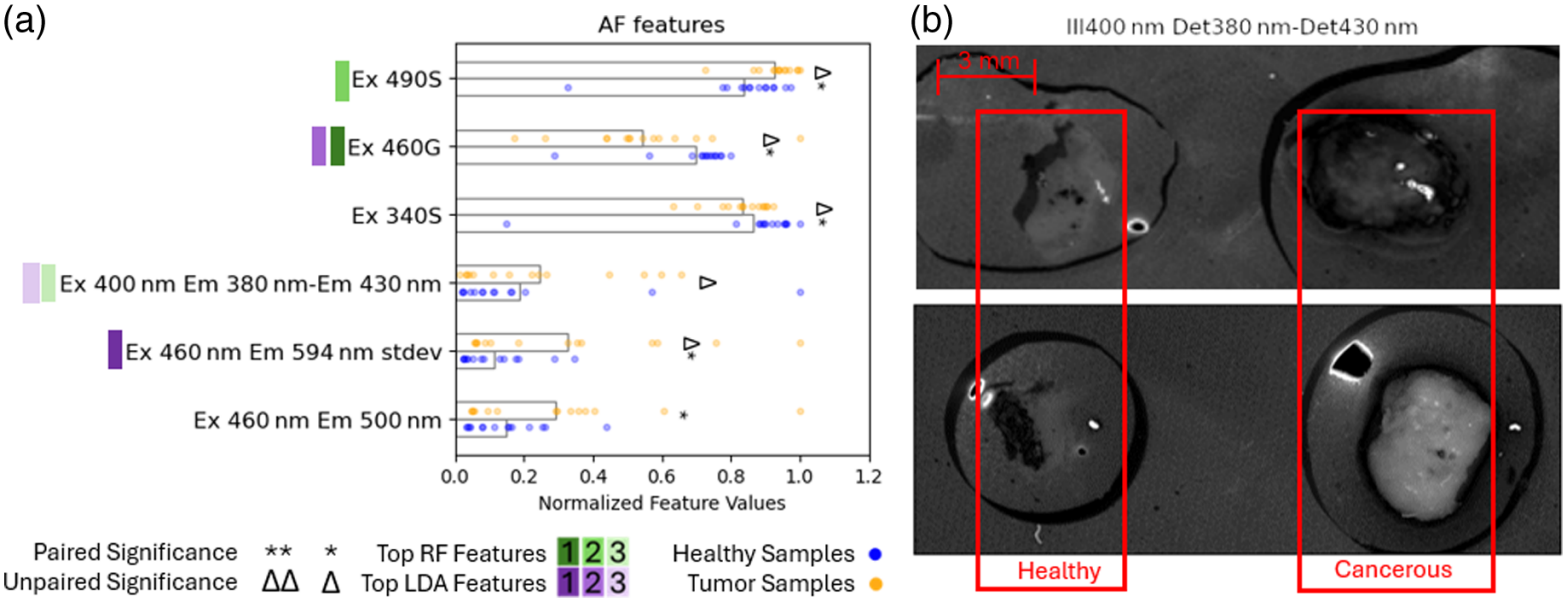
(a) Autofluorescence features of interest; for visualization purposes, the normalized values of the data points for each feature are shown with the bar lengths representing the normalized means across all samples. Significance and rank are denoted as described in Sec. 2.3.4. (b) Two different sets of sample data shown for the 400-nm illumination 380- to 430-nm feature with healthy samples on the left and cancerous on the right.

There are a number of significant AF features, shown in Fig. 5(a), particularly with excitation wavelengths between 400 and 500 nm. The paired and unpaired significance tests both resulted in five significant features with adjusted p-values below 0.1, with one feature differing for each. Of the significant features, the S and G terms show promise, as well as the simulated narrowband images. There is one standard deviation feature and one mean feature found to be significant, both with 460-nm illumination but different emission filters. The wavelengths that show significant differences between healthy and cancerous tissues can be compared with those used for narrowband imaging, which are typically 415 nm and 540  nm±30  nm to boost contrast between mucosa and vascular components.^39^^,^^40^

In examining some of the data from one of the highest-ranked features, the simulated narrowband image between 380- and 430-nm emission bands, with 400-nm excitation illumination, is shown in Fig. 5(b) for two different sample sets. We see in one case that the healthy sample has higher intensity, but the tumor is brighter in the other set. Note that in our image process pipeline, any saturated regions of the image are excluded. Despite the number of significant features found here, it can be a challenge to find sets of feature data that consistently behave similarly for one tissue type compared with another. This could be due to the differences in tissue sample size and depth of biopsy as the presence of stromal tissue in the sample which underlays the mucosa contains more collagen which is known to be fluorescent under illumination by wavelengths around 400 nm in esophageal tissue.^41^

### 3.2 Hyperspectral Data

**Fig. 6:**
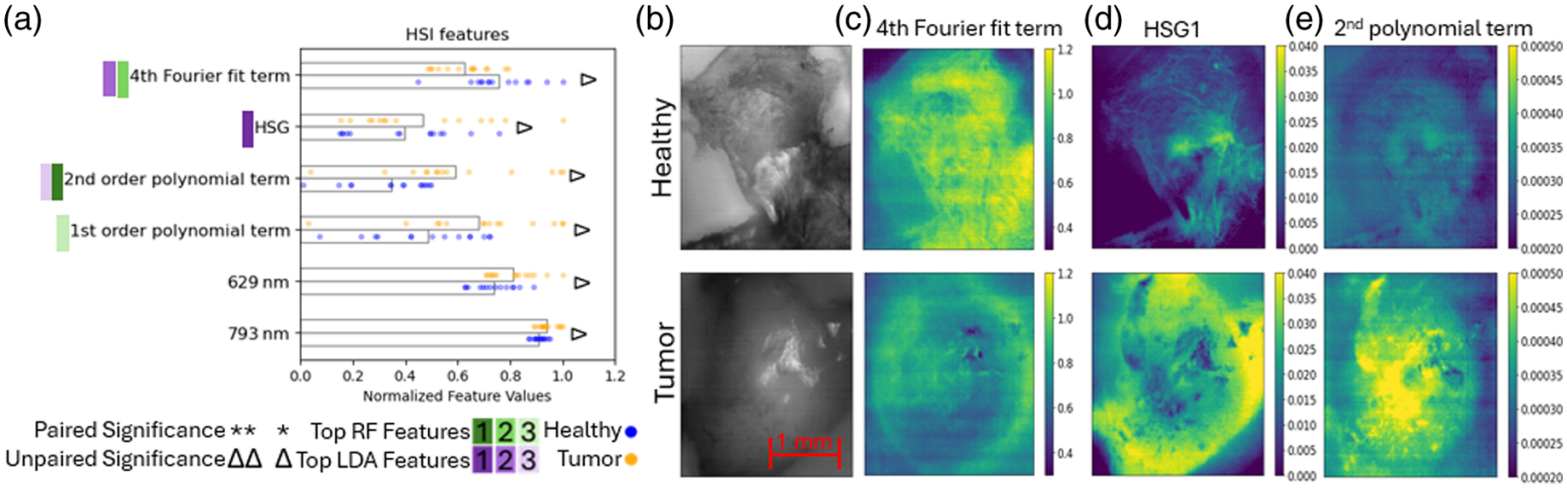
(a) Hyperspectral features of interest; for visualization purposes, the normalized values of the data points for each feature are shown with the bar lengths representing the normalized means across all samples. Significance and rank are denoted as described in Sec. 2.3.4. (b) 532-nm images taken of a healthy (top) and cancerous (bottom) sample shown for reference for features calculated over 10×10  pixel ROIs across the whole image for visualization purposes of the (c) fourth Fourier fit term, (d) hyperspectral G2 feature, and (e) second polynomial fit term.

For HSI, there were six features determined to be significant in the unpaired significance test, and no significant features in the paired significance test, as shown in Fig. 6(a). The features that ranked the highest are all frequency terms and fit parameters, which is consistent with what we observe in the data, as there were no wavelength regions of the spectral data where one tissue type had consistently different spectra. Despite this, there are two wavelength bands that show significance: 629 and 793 nm. When examining the visualizations of the data with features calculated on 10 × 10 pixel squares within the image [Figs. 6(b)–6(e)], there are some distinct differences that emerge between the two samples; however, these do not seem to be highly consistent across all samples. Even within a single specimen, the feature values do not seem to be highly consistent across the sample, in particular for the tumor sample in Figs. 6(d) and 6(e). This may suggest there could be some tissue inhomogeneity that is contributing significant variability to the data.

### 3.3 Optical Coherence Tomography Data

**Fig. 7:**
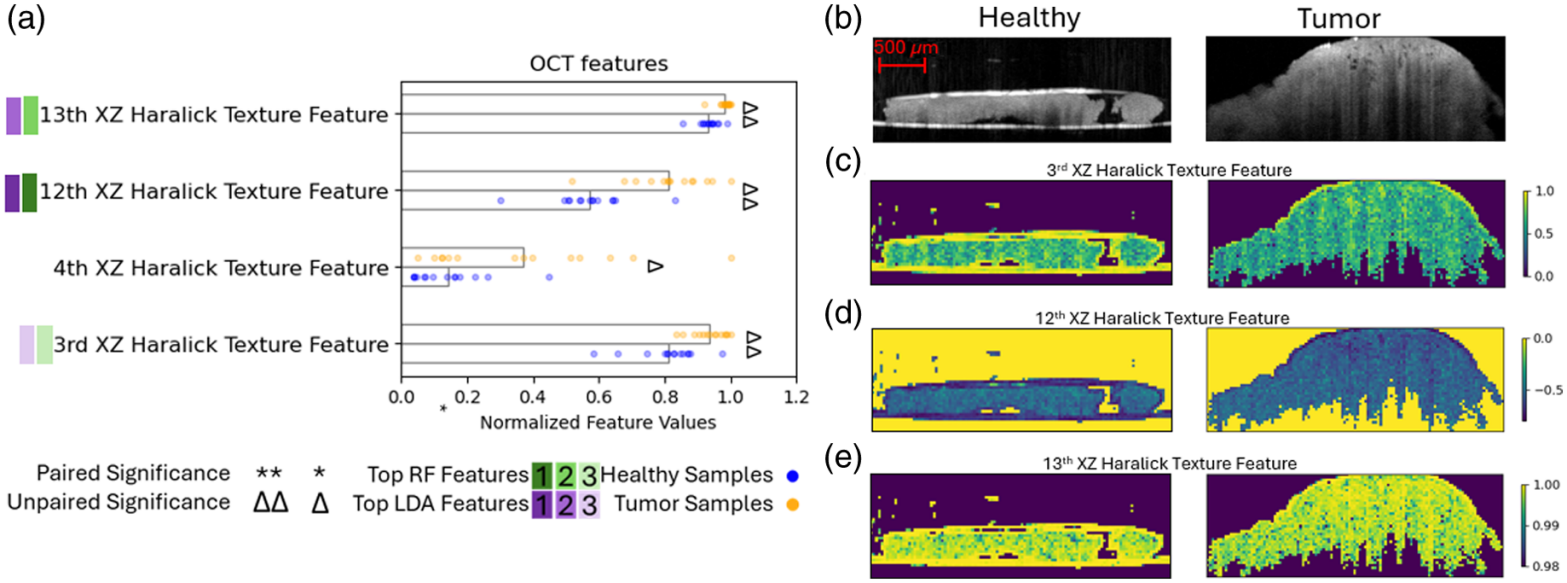
(a) Optical coherence tomography features of interest; for visualization purposes, the normalized values of the data points for each feature are shown with the bar lengths representing the normalized means across all samples. Significance and rank are denoted as described in Sec. 2.3.4. (b) Images taken of a selected slice in the XZ plane of healthy (left) and cancerous (right) samples shown for reference for features calculated over 10×10  pixel ROIs across the whole image for visualization purposes of the (c) 3rd Haralick texture feature, (d) 12th Haralick texture feature, and (e) 13th Haralick texture feature.

There were four features from the OCT dataset, which showed significance in the unpaired test, three with adjusted p-values below 0.05. These were among the three highest ranked features by the Gini importance and scatter coefficients as well, as shown in Fig. 7(a). All of these significant terms come from the XZ plane—a vertical slice through the sample. This could be due to the fact that the OCT system has higher resolution in Z than it does in X and Y, or perhaps there is more differentiating information through the tissue cross-section than in-plane. When visually examining these features calculated over 10×10  pixel squares from the samples shown in Fig. 7(b), with the resulting feature maps shown in Figs. 7(c)–7(e), there are no major noticeable visual differences between the two tissue types that clearly emerge. There are some regions in the tumor sample in Fig. 7(b) where the tissue appears more inhomogeneous, which are somewhat visible in the feature maps; however, these types of features do not appear in all tumor samples.

### 3.4 Polarized Light Imaging Data

**Fig. 8:**
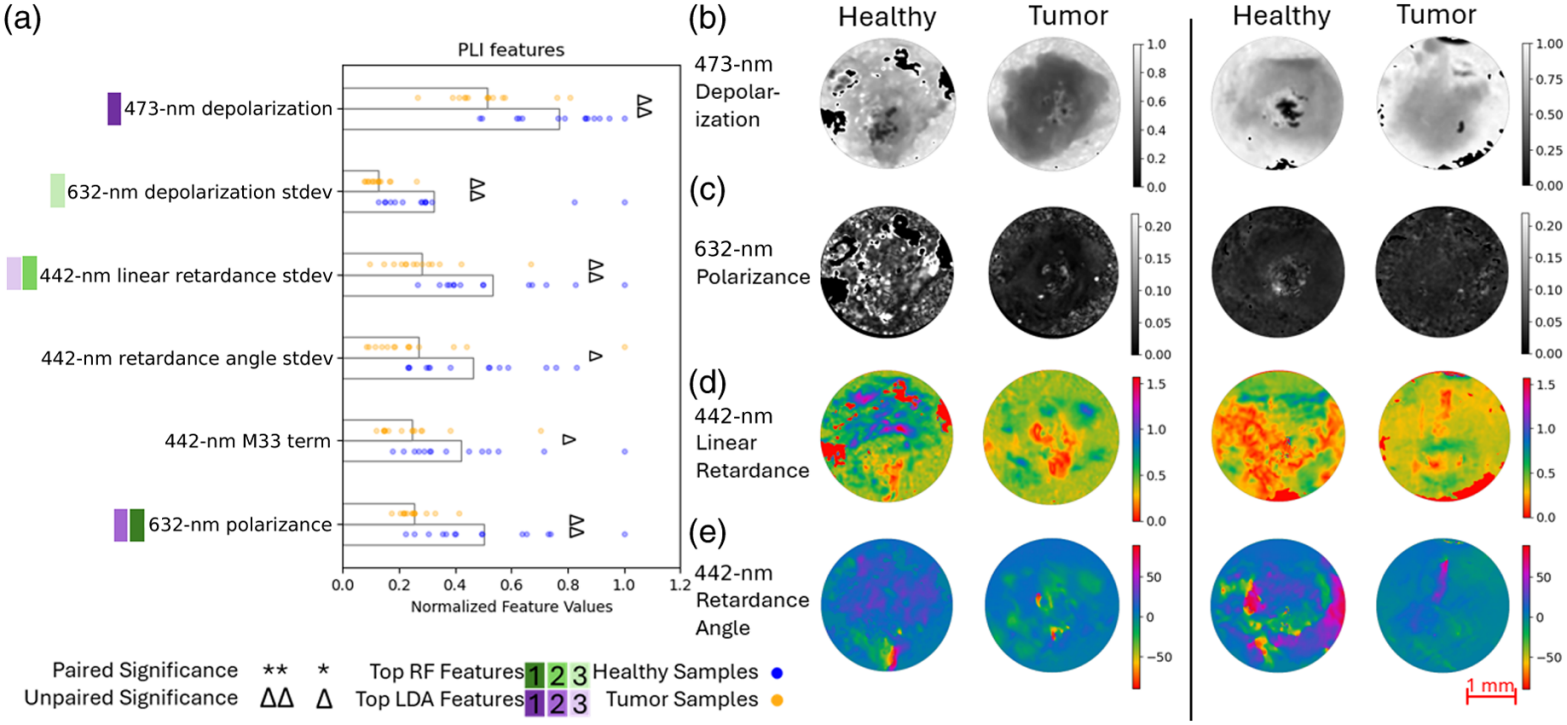
(a) Polarized light imaging features of interest; for visualization purposes, the normalized values of the data points for each feature are shown with the bar lengths representing the normalized means across all samples. Significance and rank are denoted as described in Sec. 2.3.4. (b)–(d) Two sets of samples taken from the same patient showing the (b) 473-nm depolarization, (c) 632-nm polarizance, (d) 442-nm linear retardance in radians, and (e) 442-nm retardance angle in degrees.

As shown in Fig. 8(a), PLI has the most significant features out of the four imaging modalities with adjusted p-values below 0.05, all of which were found significant in the unpaired comparison test. This could be due to more robust calibration methods built into the PLI processing software than other methods, or the structural differences that PLI is probing are more consistent from sample to sample. Among the significant terms, those related to retardance and depolarization appear to be the most prominent; these have been previously cited as polarization properties which can aid in differentiating between healthy and cancerous tissues.^18^ One of the Mueller matrix terms was found to be significant; however, these are somewhat more challenging to interpret, as many of the Mueller matrix terms contribute to multiple different polarization properties. There are several standard deviation features that show significance: this could be due to the differences in the homogeneity of the signal coming from healthy and cancerous tissue. In examining the feature maps in Figs. 8(b)–8(e), there are some clear differences between the two tissue types for each patient; however, as with the results from other modalities, they were not necessarily consistent across all samples.

### 3.5 Classification Results

**Fig. 9:**
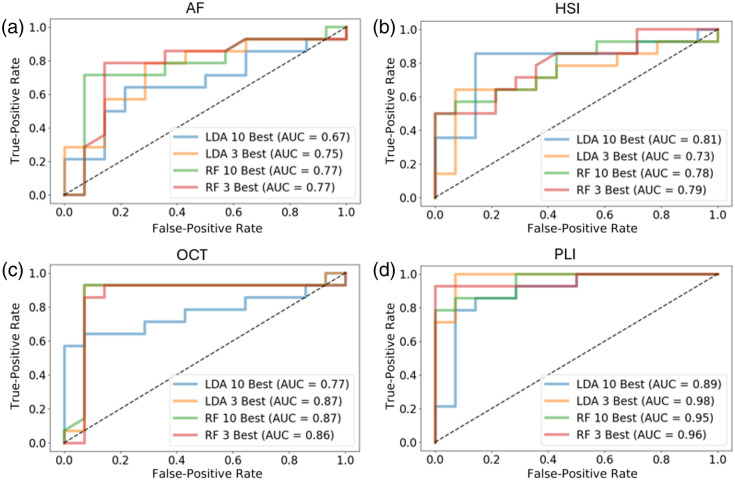
For (a) AF, (b) HSI, (c) OCT, and (d) PLI systems, the classifier performances are visualized when using features from the different imaging types. For each, ROC curves are drawn using the 3 and 10 best features ranked by scatter coefficients in an LDA classifier, and the 3 and 10 best features ranked by Gini importance in a random forest classifier.

The results of running the LDA and RF classifier with the top three features and the top 10 features for each imaging type are shown in the ROC curves in Figs. 9(a)–9(d). The AUC serves as a metric for how well the classifier is performing, with 1 being a perfect classifier and 0.5 suggesting the classifier is no better than random chance. Out of the four imaging types, PLI performs the best, followed by OCT and AF with HSI somewhat below those. These results seem to mirror the number of unpaired significant features with adjusted p-values below 0.05. In considering the two different types of classifiers and the number of features they use, in almost all cases, the two classifiers perform similarly, though LDA with 10 features typically performs worse. Other than with the HSI data, the LDA performs better with 3 features than 10, which is consistent with previous work that shows LDA typically performs better with fewer features to avoid overfitting,^25^ whereas the RF AUC values are similar for both 3 and 10 features.

### 3.6 Multimodal Imaging

**Fig. 10:**
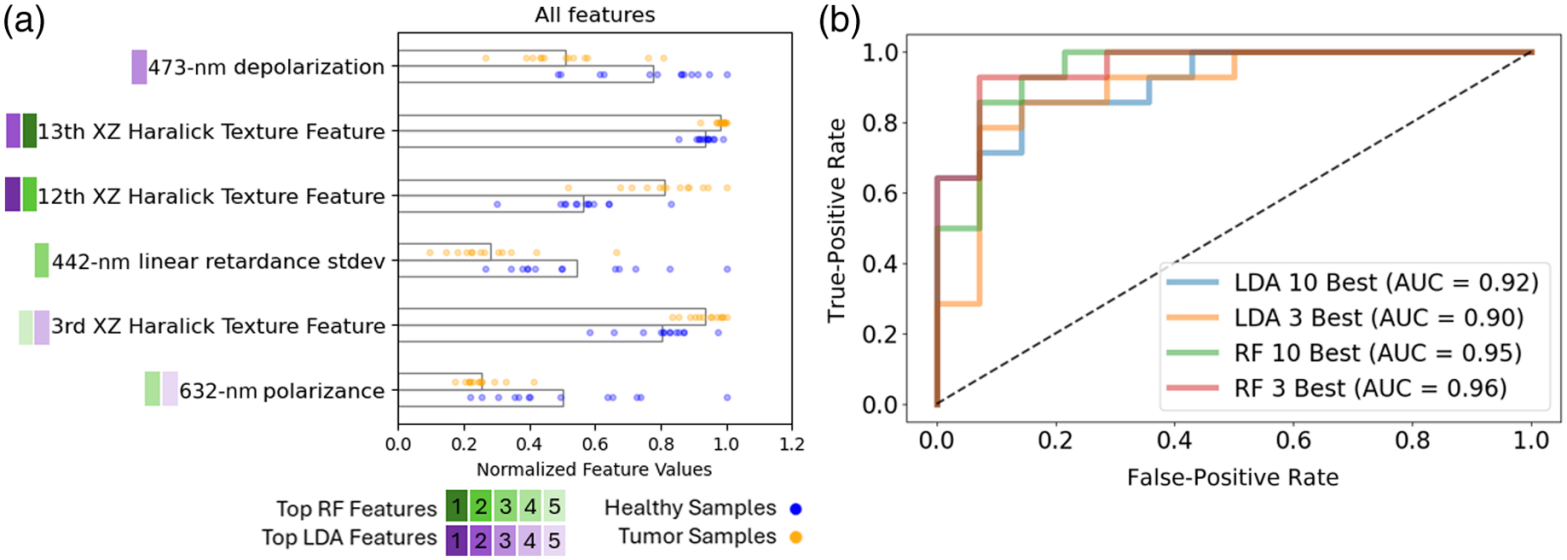
(a) Top five features from all modes; for visualization purposes, the normalized values for each feature are shown with the bar lengths representing the normalized means across all samples. Feature rank is denoted by the hue to the left of the feature name. (b) ROC curves describing the classifiers’ utility when using the best 3 and 10 best features in LDA and RF classifiers.

In assessing how the different imaging modes could complement each other, the feature ranking process was repeated with all the imaging features pooled together. The top five RF and LDA features are shown in Fig. 10(a). The classifier models are run once again using the 3 and 10 best features as determined by the scatter coefficients and Gini importance, all of which were from the PLI and OCT systems, aside from one AF and one HSI feature in the 10 best Gini importance features. As shown in Fig. 10(b), the combined features perform better than AF, HSI, and OCT and roughly equivalent to PLI. The prominence of PLI and OCT features in the best feature ranking suggests that polarization-sensitive OCT could be a fruitful avenue for future study. Both PLI and OCT probe structural elements of the tissue, whereas AF and HS focus on biochemical changes, suggesting that in this *ex vivo* context, structural changes may be more detectable. However, this approach is purely assessing the ability to discriminate tissue types mathematically with extracted feature values. Other reasons for pursuing multimodal imaging devices focusing on different functionalities such as complementary fields of view, depth penetration, and resolution are all valuable motivations as well. Overall, these results suggest that PLI has the highest potential for discriminating tissue types of these four modes and warrants further investigation for esophageal and other gastrointestinal cancer detection.

In considering how the classifiers perform when comparing different tissue types, the results can be examined in Fig. 16. In comparison with differentiating between normal and cancerous tissues, the classifier worked similarly well when comparing both the cancer and metaplasia samples to normal as seen in Fig. 16(a), slightly better when looking at normal versus metaplasia tissue, and slightly worse when just comparing cancerous and metaplasia tissue samples. These results suggest that differentiating between normal and abnormal tissues is a more straightforward challenge than differentiating between cancerous and metaplasitc tissues.

Each imaging mode generates extensive data that broadly characterize the tissue. Therefore, to facilitate the incorporation of any of these modes into a clinical endoscope, it is important to determine an ideal subset of image features to minimize complexity. The polarimeter used in this study measures the Mueller matrix across five imaging wavelengths, and our results suggest there are several wavelengths that could be beneficial with the most significant features imaged with 442- and 632-nm illumination, so the imaging time and system size could be reduced by designing a system which only uses one of these wavelength. Similarly, retardance and depolarization seem to be the most dominant polarization properties in these results, so a full Mueller polarimeter likely is not necessary for esophageal cancer detection. Ultimately, the implementation of multimodal optical imaging involves significant design and cost-of-production considerations that need to be weighed against the benefits gained.

Multimodal imaging may also offer benefits in more complex imaging scenarios than presented in this paper, such as investigating transition zones among tissue types, for example, defining the margins of tumors, where more complex features are present. In this case, significant heterogeneity in tissue architecture and composition can be observed, and a multimodal approach could help characterize the larger heterogeneity in features. Other roles where multimodal imaging could be impactful include when cancer presents with different subtypes that may exhibit significantly different characteristics. The multimodal approach may offer sensitivity to different disease subtypes through a variety of different contrast mechanisms.

### 3.7 Study Limitations and Future Directions

There are some clear limitations to this study that are important to highlight. First, working with excised tissues implies that there is no blood present in the tissue, and in general, *ex vivo* imaging is performed under very different conditions than *in vivo* endoscopy. It has been discussed previously that the spectral signature of tissue may change when collected *ex vivo*,^13^ which could help to explain why HSI and AF classification results did not perform as well as PLI and OCT, despite promising results in other past studies. Similarly, in the OCT data, there is a lack of clear layers present which may be observed with *in vivo* data. That is because the thin specimens are mostly composed of the epithelial layer of the esophagus, so the diagnostic features we are assessing are focused on the density and texture of that tissue rather than striations of the layers and larger scale architecture which have been utilized in helping to classify tissue types previously.^42^ Our feature extraction method was selected to investigate pixel-wise texture given the lack of overall tissue architecture features that are recapitulated in our small specimens.

As acknowledged above, it is not clear how well *ex vivo* findings will translate to *in vivo*, especially for biochemically sensitive techniques such as AF and HSI,^43^ and a direct comparison of different imaging modes *in vivo* versus *ex vivo* would be a rich area of future study. There have been numerous studies which have looked at the autofluorescent properties of esophageal tissue *in vivo* through endoscopic devices;^10^^,^^44^^–^^48^ however, the results and conclusions are somewhat varied among them. Georgakoudi et al.^47^ found there to be distinct differences in the fluorescence spectra when esophageal tissue was illuminated with 337 and 397 nm *in vivo* which allowed them to differentiate among different levels of dysplasia in Barrett’s esophagus regions. Curvers et al.^10^ incorporated AF into an endoscope to assess how well it would aid in screening, and they utilized blue excitation with wavelengths between 390 and 470 nm to target changes in green reflectance with wavelengths between 500 and 630 nm *in vivo*. AF helped to identify suspicious regions but did not significantly improve the screening procedure. Holz et al.^48^ found significant differences in the fluorescence spectra of esophageal tissue measured *in vivo* when illuminated with 395, 405, and 410 nm, between metaplasia and high-grade dysplasia/early cancer with a sensitivity and specificity of 80.0% and 89.5% respectively. In classification, their AUC was 0.85, which is relatively comparable to the results in this study when making a similar comparison, as in Fig. 16(c). A common thread among many of these is the use of blue light illumination and a reduction of green light in suspicious regions. Though there are two significant features in this work with illumination in similar wavelength ranges, and one that utilizes the pseudo spectrum technique that could be picking up changes in the green spectrum range, most of the significant features have wavelengths higher than those typically utilized in these *in vivo* endoscopic devices. Given the wavelength dependence of depth penetration, these differences may be attributed to the *ex vivo* nature of our specimens.

In comparing hyperspectral *in vivo* against *ex vivo*, Yoon et al.^14^ imaged 12 esophageal samples *ex vivo* fresh with a hyperspectral endoscope and found there to be significant differences in the spectral properties of esophageal cancer compared with healthy tissue and Barrett’s esophagus using a spectral angle measurement which measures divergence from the average spectra of adenocarcinoma. Qiu et al.^15^ imaged 57 patients’ esophaguses *in vivo* with a multispectral endoscopy system, looking at spectral differences in the 600- to 800-nm spectral range, reporting 88% sensitivity and 91% specificity in identifying dysplasia. Waterhouse et al.^16^ were able to endoscopically image 20 patients with a spectral endoscope, targeting differences in the vascularization, and with this, they were able to develop a machine learning algorithm that could differentiate between Barrett’s esophagus and neoplasia with an 83.7% sensitivity and an 85.5% specificity. The features that were determined to be most significant in the latter two studies are somewhat in agreement with the results reported in this paper, in that the feature primarily focused on the 600- to 800-nm spectral range and, beyond that, features that characterize the spectrum shape as a whole were the most promising. Given the wide range of feature extraction techniques available, developing the most robust and reproducible features for this technology remains a rich area of future work.

In addition, there is some selection bias in that biopsied tissue was detectable by clinicians, whereas the downstream clinical need is to improve imaging techniques and find early cancerous or dysplastic lesions that would be less likely to be detected. However, there were discrepancies in what the clinician believed the tissue to be and what the pathologist identified in the stained and sectioned slides, with 10 out of 45 of the samples being originally miscategorized—the distribution of these can be seen in Table 2. Seven out of 10 of these were samples believed to be cancerous tissue by the clinician, which were determined to be normal or metaplasia by the pathologist. At a more practical level, the small size of the samples also posed challenges in ensuring orientation was consistent for imaging across all samples. Although every effort was made to ensure the epithelial side of the tissue was facing toward the imaging systems, there was sometimes a lack of discernible features that could be used for reference. This may introduce some errors, and it is worth noting that due to the small size of the samples, many specimens were observed to be relatively homogeneous throughout the depth during pathological inspection—particularly the tumor specimens—which would mitigate issues associated with any orientation errors. Future work involving imaging larger specimens (e.g., surgical specimens) would provide a means to verify our results in tissues more reflective of *in vivo* architecture.

Another important consideration to investigate is how tissue alterations that are not specific to disease, such as inflammation, may influence the relevance and performance of imaging features and classifiers. Inflammation is known to play a significant role in the development of esophageal cancer, as well as associated conditions,^49^ and inflammatory markers can influence light-tissue interaction that may mimic disease-related changes, which could result in false positives or false negatives in a classifier based on features derived from tissues without accounting for this factor. The multimodal approach may offer an avenue to address the influence of inflammation by providing simultaneous normalization or calibration features, as well as diagnostic features using contrast from combined modalities.

Currently, the data processing pipeline implemented here does not perform co-registration among imaging types as features are extracted and averaged over a spatial region of interest. The drawback of this approach is that it may not capture spatially varying features within our specimens, which may limit the performance in the presence of significant heterogeneity. A rich area of future work would be exploring spatially resolved feature mapping through co-registration across modalities to assess if there are correlations in how the features vary across the samples. Co-registration would also enable easier implementation of machine learning algorithms that operate with two- or three-dimensional image input, such as convolutional neural networks. In future iterations of this work, feature extraction procedures could be assisted by applying machine learning algorithms for unsupervised feature extraction or direct end-to-end classification. In this work, we focus on handcrafted image features more clearly tied to the optical properties of the tissue as these retain a higher level of explainability and interpretability, but developing novel machine learning methods to analyze multimodal data could provide significant improvements in accuracy and robustness. Indeed, the development of multimodal deep learning algorithms has recently gained significant interest.^50^ This study focuses primarily on identifying features with significant differences among normal tissue, metaplasia, and cancer and also those that offer high predictive ability in classifiers. Although our features were selected with direct interpretability in mind, there is still the limitation that these features will not likely offer significant intuitive guidance for end users as extracted values; therefore, future applications would likely involve integrating these features into an end-to-end tool for tissue analysis and identification of diseased or suspicious areas. Visualizations of these features could be generated as a clinical guidance tool similar to those in Figs. 5–8. Nonetheless, we believe this to be a valuable first step in assessing these imaging modes for further development and better understanding of those differences and biases.

## 4 Conclusion

This study collected 45 fresh human esophageal tissue samples: 20 healthy, 14 cancerous, and the remaining classified as other forms of metaplasia. These samples were imaged using four modalities—AF, HSI, OCT, and PLI—to identify the most promising contrast mechanisms for distinguishing between the two tissue types and to evaluate the potential for a multimodal endoscopic system. After imaging, key features were extracted from the datasets for each sample, which were statistically evaluated and used in classification algorithms to measure their utility in distinguishing between healthy and cancerous tissues. Out of the four imaging modes, PLI shows the most promise for differentiating among the sample types, in particular the retardance and depolarization terms seem to be the most significant in assessing differences between the two. In our results, we see slight improvements in the performance of tissue classifiers when using multimodal data compared with single modality, in particular with PLI and OCT. Beyond this, multimodal imaging with these modes could provide valuable functionality for biomedical purposes, for example, providing multiple scales of field of view, resolution, and depth penetration. Although the results in this publication do not show distinct statistical advantages to incorporating multiple imaging modes into an endoscopic system, this extended functionality could be highly advantageous for the challenges of highly variable *in vivo* imaging conditions, which warrants further study.

Beyond the data discussed in this paper, Barrett’s esophageal samples were collected when available as well as patient demographic metadata and RNA sequencing data. In the future, we hope to continue to expand this dataset to more closely tie the optical properties of the tissue to particular pathological changes. The work presented here lays the ground work for that by establishing imaging and data processing techniques; continuing to fine-tune methods for identifying optical biomarkers in *ex vivo* environments, tie them to *in vivo* environments and improve the specificity of data analysis procedures could be crucial in paving the way to more accurate and efficient endoscopes and concurrent diagnostic software.

## 5 Appendix

**Fig. 11:**
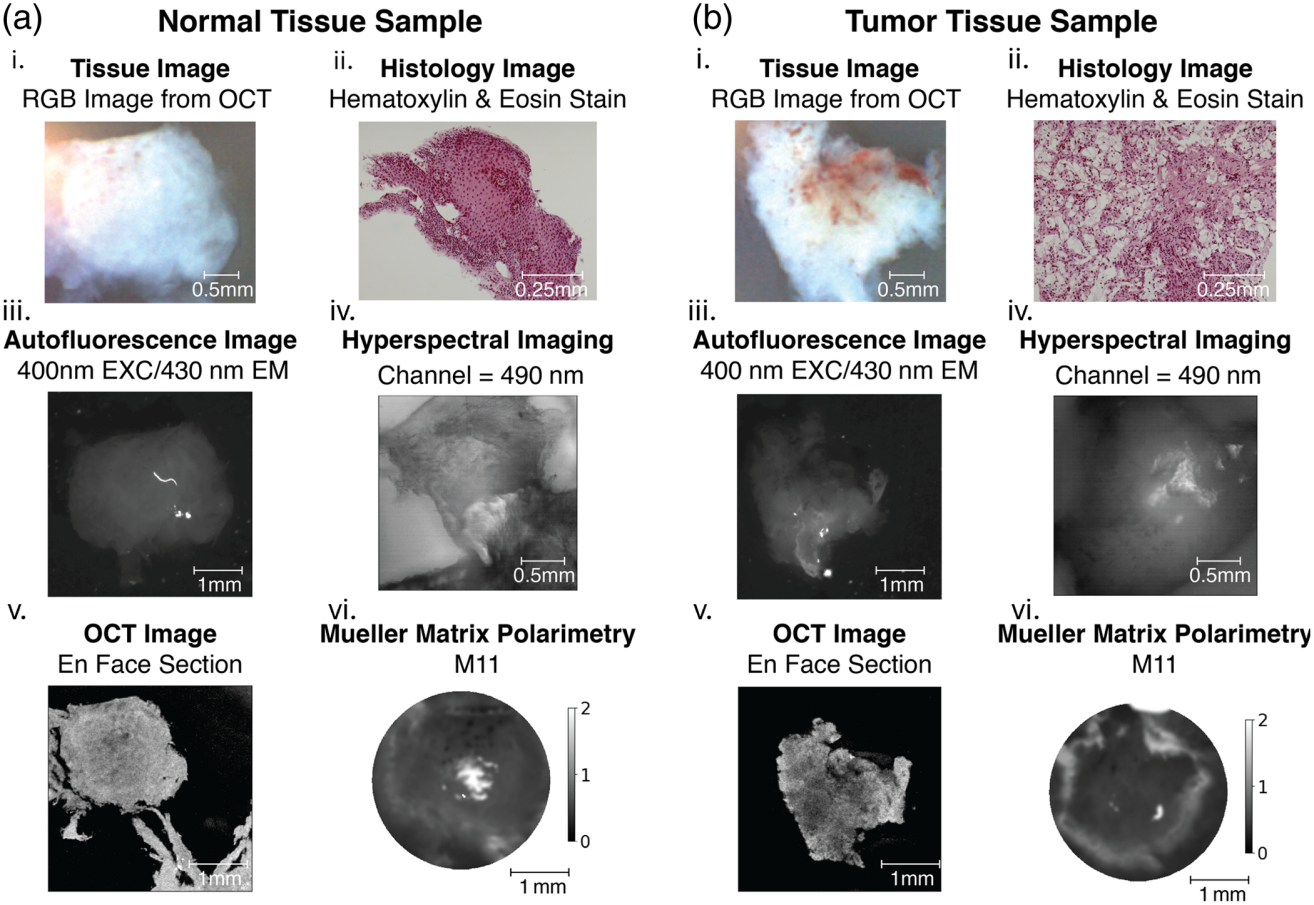
(a) Normal esophageal tissue and (b) cancerous esophageal tissue sample image data with (i) white light images of the samples, (ii) microscopy images of the samples after being H&E-stained and sectioned, (iii) images taken with the autofluorescence imaging system, (iv) images taken with the hyperspectral imaging system, (v) images taken with the optical coherence tomography system, and (vi) M11 intensity images taken with the Mueller matrix polarimeter.

**Fig. 12:**
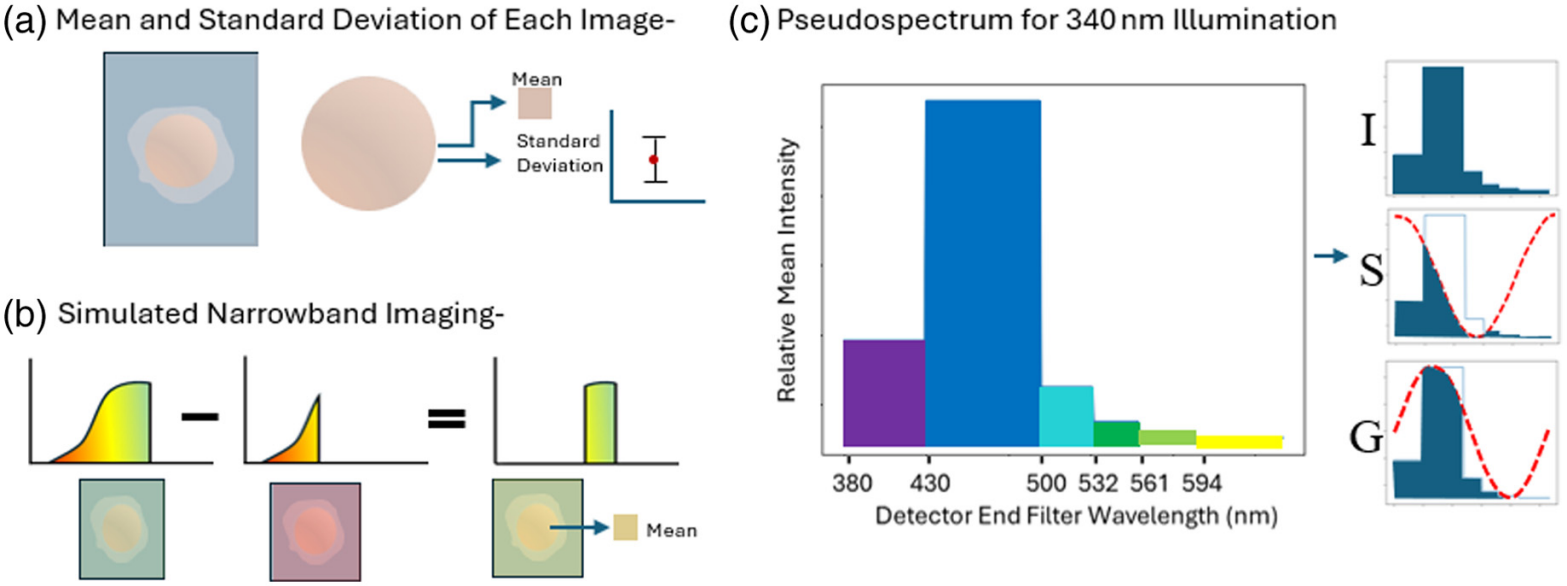
(a) Mean and standard deviation taken across ROI for each image taken. (b) Simulated narrowband images created by subtracting lower detector filters from higher for each illumination wavelength. (c) Pseudo-spectrum is calculated for each illumination wavelength by interpolating the narrowband images from here the 0^th^-order and real and imaginary components of the first-order frequency terms are taken.

**Fig. 13:**
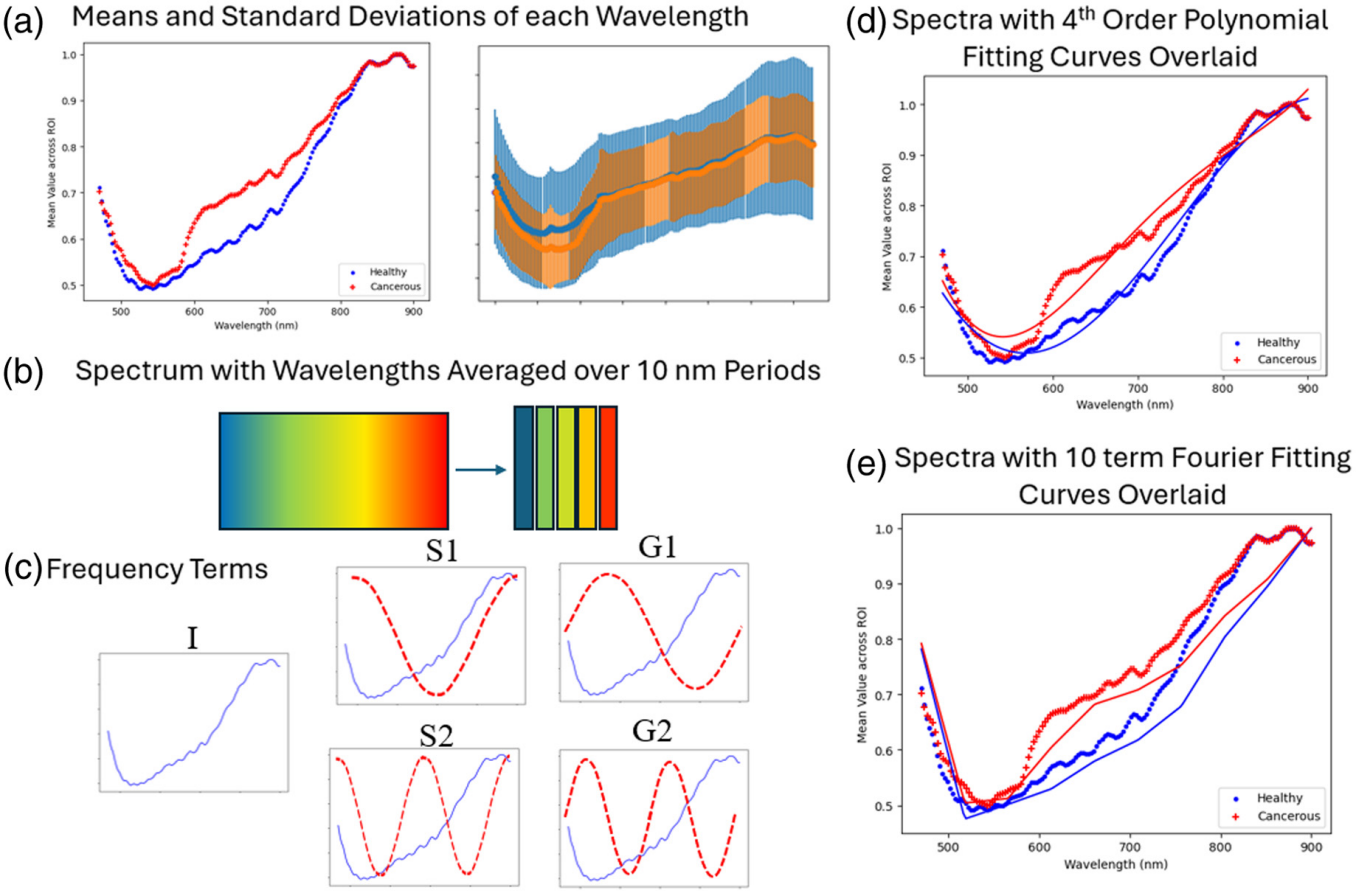
(a) Mean and standard deviation taken across ROI for each imaging wavelength. (b) Spectrum is averaged over 10-nm periods, and the mean is taken of those values. (c) The 0^th^-order and real and imaginary components of the first- and second-order frequency terms are taken. (d) Spectra formed by taking the means of the ROI over each wavelength are fit to a fourth-order polynomial curve, and the fit parameters are taken as features. (e) Top 10 Fourier frequency terms are taken as features.

**Fig. 14:**
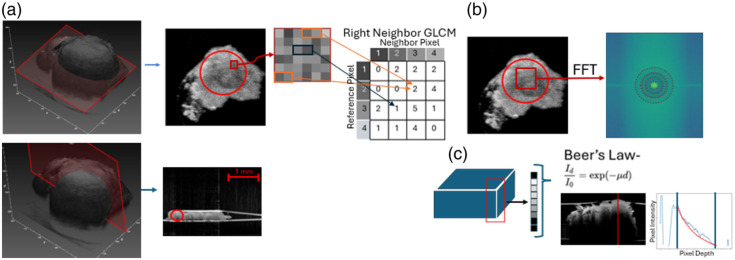
(a) Slices are taken in the XY and XZ planes, and Haralick texture feature extraction is done over the ROI. (b) FFT is taken over a consistently sized ROI for all samples from the slices, and frequency features are extracted over annular ROIs. (c) Vertical slices are taken across the sample, and values are fit to a decaying exponential to estimate the sample’s attenuation rate in accordance with Beer’s law.

**Fig. 15:**
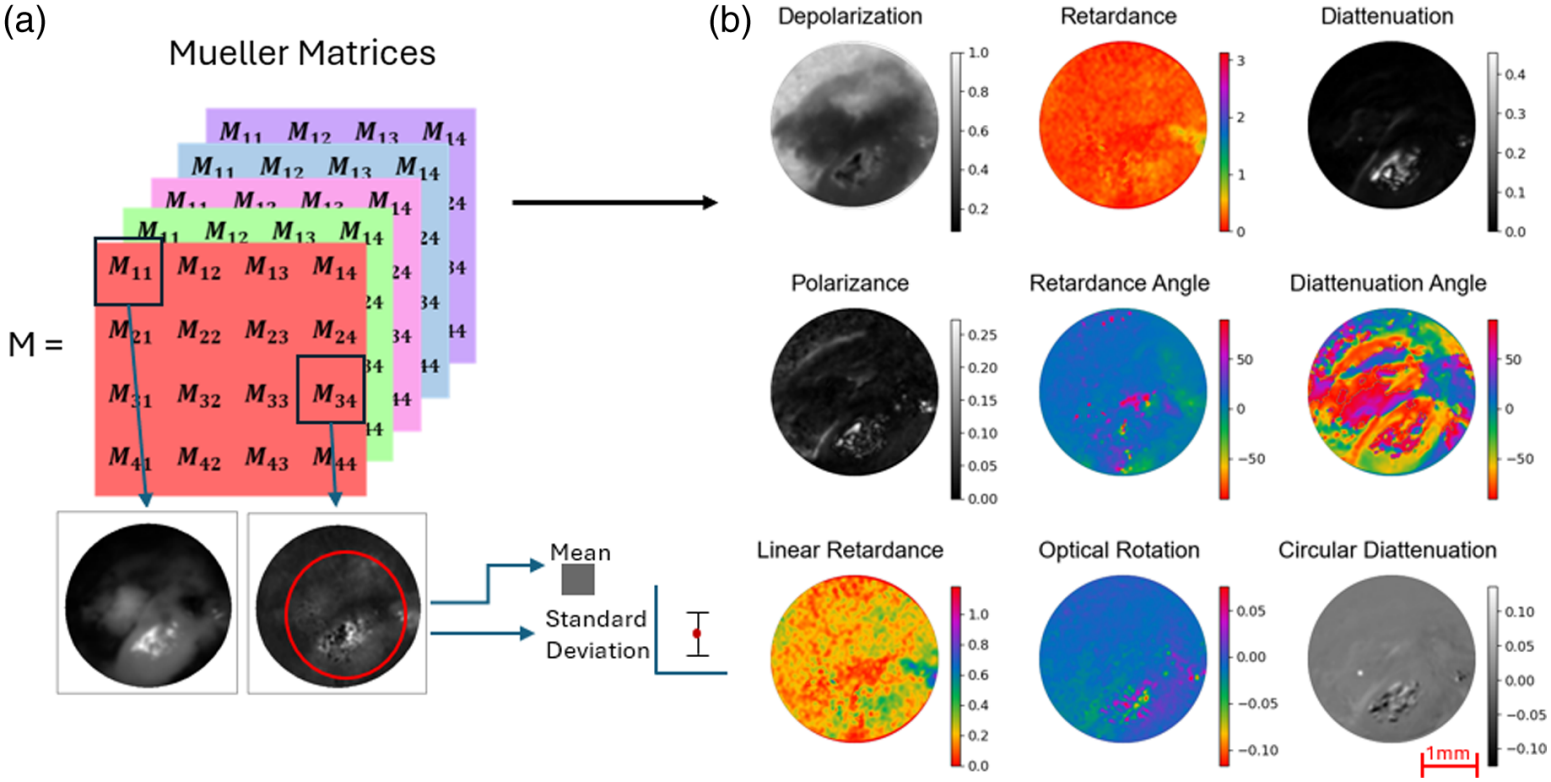
(a) Mueller matrices are calculated for each wavelength, and the mean and standard deviation are taken for each term in the Mueller matrix over an ROI within the sample. (b) Lu Chipman decomposition is applied to the Mueller matrices to isolate the effects of the different polarization properties shown here. The mean and standard deviation of each are calculated over an ROI.

**Fig. 16:**
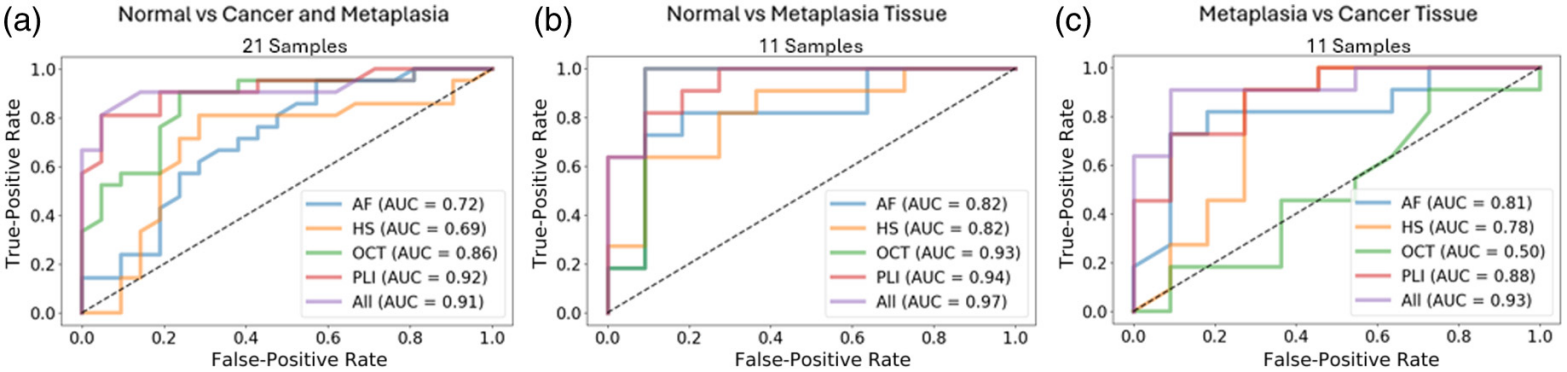
Random forest classification results for all four imaging modes and all features combined comparing (a) normal versus cancerous and metaplasia tissue grouped together, (b) normal versus metaplasia tissue, and (c) metaplasia versus cancerous tissue.

Figure 11 shows example images of normal and tumor tissue specimens, including bright-field photography and histology, as well as images from each of the label-free imaging modalities. Diagrams of feature extraction processes are shown for autofluorescence (Fig. 12), hyperspectral (Fig. 13), optical coherence tomography (Fig. 14), and Mueller matrix polarimetry (Fig. 15). Figure 16 shows results of random forest classification for tissue classified as metaplastic when compared to other tissue types. Table 2 includes a summary of pathological and clinical classification of each tissue specimen. The pathological classification was used for establishing classification labels.

**Table 2:** Tissue type of samples. The “Agree” column enumerates the samples where the pathologist and the clinician gave the tissue the same classification, the “Differ” column shows the samples where there were discrepancies between the two classifications, and the “NA” column shows the samples which the pathologist could not classify.

Tissue class	Tissue type	Agree	Differ	NA	Total	Class total
Normal	Epithelium	14	3	3	20	20
Cancer	Adenocarcinoma	7	0	0	7	
	Squamous cell carcinoma	1	0	0	1	
	Melanoma	1	0	0	1	
	Plasmacytoma	1	0	0	1	
	Not classified	0	0	4	4	14
Metaplasia	Barretts	4	2	0	6	
	Not classified	0	5	0	5	11

## Data Availability

All data used in the preparation of this publication can be found at DOI: https://doi.org/10.25422/azu.data.28207508. Concurrent code used in data processing and analysis can be found at https://github.com/travissawyer/multimodal-imaging-esophagus.
